# Monoclinic form I of clopidogrel hydrogen sulfate from powder diffraction data

**DOI:** 10.1107/S1600536810028783

**Published:** 2010-07-24

**Authors:** Vladimir V. Chernyshev, Sergey V. Pirogov, Irina N. Shishkina, Yurii A. Velikodny

**Affiliations:** a Department of Chemistry, Moscow State University, 119991 Moscow, Russian Federation; b JSC "Active Component", bld. 5A, Road to Metallostroy, Metallostroy, Saint Petersburg, 196641, Russian Federation

## Abstract

The asymmetric unit of the title compound, C_16_H_17_ClNO_2_S^+^·HSO_4_^−^, (I) [systematic name: (+)-(*S*)-5-[(2-chloro­phen­yl)(meth­oxy­carbon­yl)meth­yl]-4,5,6,7-tetra­hydro­thieno[3,2-*c*]pyridin-5-ium hydrogen sulfate], contains two independent cations of clopidogrel and two independent hydrogensulfate anions. The two independent cations are of similar conformation; however, this differs from that observed in ortho­rhom­bic form (II) [Bousquet *et al.* (2003 ▶). US Patent No. 6 504 030]. The H—N—C_chiral_—H fragment shows a *trans* conformation in both independent cations in (I) and a *gauche* conformation in (II). In (I), classical inter­molecular N—H⋯O and O—H⋯O hydrogen bonds link two independent cations and two independent anions into an isolated cluster, in which two cations inter­act with one anion only *via* N—H⋯O hydrogen bonds. Weak inter­molecular C—H⋯O hydrogen bonds further consolidate the crystal packing.

## Related literature

For the characterization of six polymorphic forms of Clopidogrel hydrogensulfate, see: Badorc & Frehel (1989 ▶) (form I); Bousquet *et al.* (2003 ▶) (ortho­rhom­bic form II); Lifshitz-Liron *et al.* (2006 ▶) (forms III-VI). For recent studies of forms I and II, see: Raijada *et al.* (2010 ▶); Zupancic *et al.* (2010 ▶); Srivastava *et al.* (2010 ▶); Song *et al.* (2010 ▶). For details of the indexing algorithm, see: Werner *et al.* (1985 ▶). The methodology of the refinement (including applied restraints and constraints) was described in detail by Chernyshev *et al.* (2009 ▶).
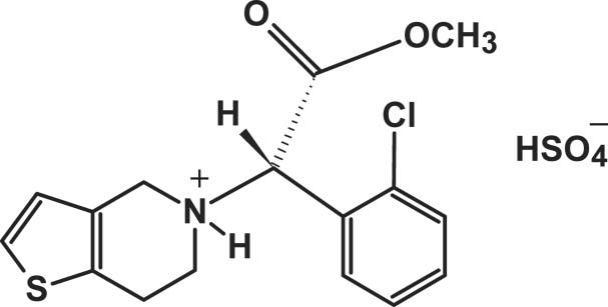


## Experimental

### 

#### Crystal data

C_16_H_17_ClNO_2_S^+^·HSO_4_^−^
*M* *_r_* = 419.89
Monoclinic, 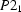

*a* = 10.4315 (12) Å
*b* = 15.3345 (18) Å
*c* = 12.6320 (16) Å
β = 113.28 (2)°
*V* = 1856.1 (5) Å^3^
*Z* = 4
Cu *K*α_1_ radiation, λ = 1.54059 Å
μ = 4.23 mm^−1^
*T* = 295 K
Flat sheet, 15 × 1 mm

#### Data collection

Guinier camera G670 diffractometer
Specimen mounting: thin layer in the specimen holder of the camera
Data collection mode: transmission
Scan method: continuous
2θ_min_ = 4.00°, 2θ_max_ = 90.00°, 2θ_step_ = 0.01°

#### Refinement

*R*_p_ = 0.019
*R*_wp_ = 0.025
*R*_exp_ = 0.015
*R*_Bragg_ = 0.049
χ^2^ = 2.982
8601 data points
205 parameters
155 restraints
H-atom parameters not refined

### 

Data collection: *G670 Imaging Plate Guinier Camera Software* (Huber, 2002 ▶); cell refinement: *MRIA* (Zlokazov & Chernyshev, 1992 ▶); data reduction: *G670 Imaging Plate Guinier Camera Software*; method used to solve structure: simulated annealing (Zhukov *et al.*, 2001 ▶); program(s) used to refine structure: *MRIA*; molecular graphics: *PLATON* (Spek, 2009 ▶); software used to prepare material for publication: *MRIA* and *SHELXL97* (Sheldrick, 2008 ▶).

## Supplementary Material

Crystal structure: contains datablocks I, global. DOI: 10.1107/S1600536810028783/lh5082sup1.cif

Rietveld powder data: contains datablocks I. DOI: 10.1107/S1600536810028783/lh5082Isup2.rtv

Additional supplementary materials:  crystallographic information; 3D view; checkCIF report

## Figures and Tables

**Table 1:** Hydrogen-bond geometry (Å, °)

*D*—H⋯*A*	*D*—H	H⋯*A*	*D*⋯*A*	*D*—H⋯*A*
N5*A*—H5*A*⋯O3*A*	0.91	1.91	2.785 (16)	161
N5*B*—H5*B*⋯O6*A*	0.91	1.94	2.795 (19)	157
O5*A*—H51⋯O6*B*	0.82	1.85	2.640 (17)	161
O5*B*—H52⋯O4*A*	0.82	1.82	2.567 (17)	152
C4*A*—H4*A*1⋯O4*B*^i^	0.97	2.35	3.17 (2)	142
C4*A*—H4*A*2⋯O1*B*	0.97	2.52	3.225 (17)	129
C3*B*—H3*B*⋯O4*B*^ii^	0.93	2.41	3.28 (2)	154
C6*A*—H6*A*2⋯O3*B*^i^	0.97	2.31	3.175 (19)	149
C4*B*—H4*B*2⋯O3*B*^iii^	0.97	2.23	3.13 (2)	154

Symmetry codes: (i) 
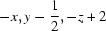
; (ii) 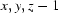; (iii) 
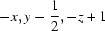
.

**Fig. 1.**
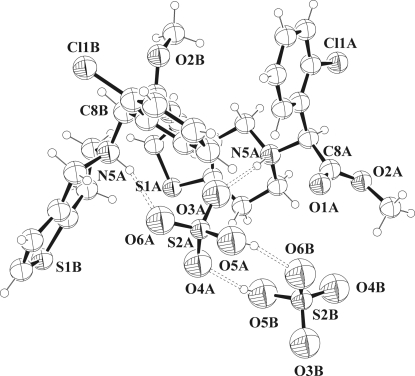
The content of asymmetric unit of I showing the atomic labeling and 40% probability displacement spheres. Dashed lines denote classical N—H···O and O—H···O hydrogen bonds.

**Fig. 2.**
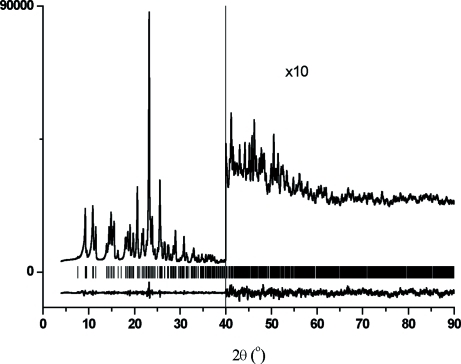
The Rietveld plot, showing the observed and difference profiles for I. The reflection positions are shown above the difference profile.

## supplementary crystallographic information

### Comment

Clopidogrel hydrogensulfate is an antiplatelet drug, which acts by selective and irreversible inhibition of ADP-induced platelet aggregation. The drug is available in the market as oral solid dosage form. Six different polymorphs are known for the drug - I (Badorc & Frehel, 1989), II (Bousquet *et al.*, 2003) and III-VI (Lifshitz-Liron *et al.*, 2006). However, only polymorphs I and II are used in pharmaceutical formulations (Bousquet *et al.*, 2003), and, therefore, they are under intensive studies (Raijada *et al.*, 2010; Zupan˘ci˘c *et al.*, 2010; Srivastava *et al.*, 2010; Song *et al.*, 2010). The crystal structure of orthorhombic polymorph II has been reported by Bousquet *et al.* (2003). Herewith we report the crystal structure of the monoclinic polymorph I.

The asymmetric unit of I (Fig. 1), contains two independent cations of clopidogrel and two independent hydrogensulfate anions. The two independent cations are of similar conformation, which, however, differs from that observed in II. The H–N—C_chiral_–H fragment shows a *trans* conformation in both independent cations in I and a *gauche* conformation in II.

The hydrogen-bonding motifs in I and II are essentially different too. In I, the classical intermolecular N—H···O and O—H···O hydrogen bonds (Table 1) link two independent cations and two independent anions into isolated cluster, where two cations interact with one anion only *via* N—H···O hydrogen bonds (Fig. 1). Weak intermolecular C—H···O hydrogen bonds (Table 1) consolidate further the crystal packing of I. In II, O—H···O hydrogen bonds link anions into linear chains, while N—H···O hydrogen bond attach one cation to one anion. These differences in crystal packings of Forms I and II may explain why II exhibits a lower solubility (and is more stable) than I.

### Experimental

The title compound I was synthesized in accordance with the known procedure (Badorc & Frehel, 1989), and obtained as a white polycrystalline powder. Optical rotation [α]_D_ +53.8° (c<ι> 1.9, CH_3_OH).

### Refinement

During the exposure, the specimen was spun in its plane to improve particle statistics. The monoclinic unit-cell dimensions were determined with the indexing program TREOR (Werner *et al.*, 1985), *M*_20_=37, using the first 30 peak positions. The same monoclinic unit-cell dimensions were reported in 2003 by Martin Vickers at http://img.chem.ucl.ac.uk/www/reports/clopi/clopi.htm.

The structure of was solved by simulated annealing procedure (Zhukov *et al.*, 2001) and refined following the methodology described in (Chernyshev *et al.*, 2009). For non-H atoms, ten independent *U*_iso_ parameters were refined - six for six independent Cl and S atoms, two common *U*_iso_ for two groups of anion' oxygen atoms, and two common *U*_iso_ for the rest atoms in independent cations. H atoms were placed in geometrically calculated positions and not refined. The diffraction profiles and the differences between the measured and calculated profiles are shown in Fig. 2.

### Crystal data

**Table:**

C_16_H_17_ClNO_2_S^+^·HSO_4_^−^	*D*_x_ = 1.503 Mg m^−^^3^
*M**_r_* = 419.89	Melting point: 455(3) K
Monoclinic, *P*2_1_	Cu *K*α_1_ radiation, λ = 1.54059 Å
*a* = 10.4315 (12) Å	µ = 4.23 mm^−^^1^
*b* = 15.3345 (18) Å	*T* = 295 K
*c* = 12.6320 (16) Å	Particle morphology: plate
β = 113.28 (2)°	white
*V* = 1856.1 (5) Å^3^	flat sheet, 15 × 1 mm
*Z* = 4	Specimen preparation: Prepared at 295 K and 101 kPa
*F*(000) = 872	

### Data collection

**Table:**

Guinier camera G670 diffractometer	Data collection mode: transmission
Radiation source: line-focus sealed tube	Scan method: continuous
Curved Germanium (111)	2θ_min_ = 4.00°, 2θ_max_ = 90.00°, 2θ_step_ = 0.01°
Specimen mounting: thin layer in the specimen holder of the camera	

### Refinement

**Table:**

Refinement on *I*_net_	Profile function: split-type pseudo-Voigt (Toraya, 1986)
Least-squares matrix: full with fixed elements per cycle	205 parameters
*R*_p_ = 0.019	155 restraints
*R*_wp_ = 0.025	42 constraints
*R*_exp_ = 0.015	H-atom parameters not refined
*R*_Bragg_ = 0.049	Weighting scheme based on measured s.u.'s
χ^2^ = 2.982	(Δ/σ)_max_ = 0.001
8601 data points	Background function: Chebyshev polynomial up to the 5th order
Excluded region(s): none	Preferred orientation correction: none

### Special details

**Table:**

Geometry. All e.s.d.'s (except the e.s.d. in the dihedral angle between two l.s. planes) are estimated using the full covariance matrix. The cell e.s.d.'s are taken into account individually in the estimation of e.s.d.'s in distances, angles and torsion angles; correlations between e.s.d.'s in cell parameters are only used when they are defined by crystal symmetry. An approximate (isotropic) treatment of cell e.s.d.'s is used for estimating e.s.d.'s involving l.s. planes.

### Fractional atomic coordinates and isotropic or equivalent isotropic displacement parameters (Å^2^)

**Table:**

	*x*	*y*	*z*	*U*_iso_*/*U*_eq_	
Cl1A	0.4095 (4)	0.6092 (3)	1.1269 (4)	0.0731 (15)*	
S1A	−0.2971 (4)	0.6556 (3)	0.6216 (4)	0.0642 (15)*	
O1A	0.2960 (8)	0.9086 (7)	0.9643 (8)	0.065 (3)*	
O2A	0.2551 (8)	0.8678 (6)	1.1168 (7)	0.065 (4)*	
C2A	−0.2033 (15)	0.5796 (11)	0.5834 (13)	0.066 (6)*	
H2A	−0.2406	0.5448	0.5181	0.079*	
C3A	−0.0682 (14)	0.5760 (10)	0.6594 (12)	0.065 (6)*	
H3A	−0.0030	0.5365	0.6547	0.078*	
C4A	0.1068 (13)	0.6546 (11)	0.8382 (12)	0.065 (5)*	
H4A1	0.1257	0.6125	0.8999	0.078*	
H4A2	0.1751	0.6463	0.8048	0.078*	
N5A	0.1194 (10)	0.7458 (9)	0.8865 (9)	0.065 (4)*	
H5A	0.1212	0.7831	0.8310	0.078*	
C6A	−0.0077 (15)	0.7685 (9)	0.9103 (12)	0.066 (6)*	
H6A1	0.0063	0.8255	0.9464	0.079*	
H6A2	−0.0161	0.7266	0.9647	0.079*	
C7A	−0.1428 (14)	0.7696 (10)	0.8044 (12)	0.065 (6)*	
H7A1	−0.2214	0.7728	0.8269	0.078*	
H7A2	−0.1455	0.8201	0.7574	0.078*	
C8A	0.2536 (14)	0.7586 (10)	0.9906 (12)	0.065 (6)*	
H8A	0.2491	0.7262	1.0559	0.078*	
C9A	0.3733 (14)	0.7242 (10)	0.9634 (12)	0.065 (6)*	
C10A	0.4519 (12)	0.6534 (11)	1.0198 (12)	0.065 (6)*	
C11A	0.5690 (13)	0.6242 (10)	1.0020 (12)	0.065 (6)*	
H11A	0.6223	0.5766	1.0406	0.078*	
C12A	0.5986 (14)	0.6727 (11)	0.9213 (12)	0.065 (6)*	
H12A	0.6703	0.6529	0.9013	0.078*	
C13A	0.5311 (13)	0.7470 (11)	0.8691 (12)	0.065 (5)*	
H13A	0.5617	0.7792	0.8213	0.078*	
C14A	0.4160 (14)	0.7729 (9)	0.8893 (12)	0.065 (6)*	
H14A	0.3669	0.8226	0.8536	0.078*	
C15A	0.2739 (15)	0.8538 (10)	1.0203 (12)	0.065 (5)*	
C16A	0.2910 (14)	0.9531 (10)	1.1684 (13)	0.066 (6)*	
H16A	0.2731	0.9553	1.2373	0.098*	
H16B	0.2357	0.9964	1.1151	0.098*	
H16C	0.3881	0.9642	1.1873	0.098*	
C17A	−0.1513 (14)	0.6881 (10)	0.7374 (12)	0.065 (6)*	
C18A	−0.0391 (13)	0.6396 (10)	0.7466 (12)	0.065 (6)*	
Cl1B	0.4361 (4)	0.7397 (3)	0.3437 (4)	0.0692 (14)*	
S1B	−0.3489 (4)	0.8614 (3)	0.1741 (4)	0.0670 (16)*	
O1B	0.1492 (9)	0.6692 (7)	0.5994 (8)	0.072 (4)*	
O2B	0.3318 (9)	0.6156 (6)	0.5793 (8)	0.072 (4)*	
C2B	−0.2713 (14)	0.9258 (11)	0.1059 (12)	0.072 (6)*	
H2B	−0.3189	0.9664	0.0493	0.086*	
C3B	−0.1318 (13)	0.9106 (12)	0.1440 (12)	0.072 (6)*	
H3B	−0.0732	0.9378	0.1146	0.087*	
C4B	0.0592 (15)	0.8170 (11)	0.2903 (13)	0.072 (6)*	
H4B1	0.1222	0.8665	0.3124	0.086*	
H4B2	0.0822	0.7819	0.2363	0.086*	
N5B	0.0783 (11)	0.7634 (8)	0.3955 (10)	0.072 (5)*	
H5B	0.0669	0.8006	0.4472	0.086*	
C6B	−0.0383 (15)	0.6974 (10)	0.3650 (13)	0.072 (6)*	
H6B1	−0.0191	0.6578	0.4293	0.086*	
H6B2	−0.0411	0.6634	0.2993	0.086*	
C7B	−0.1796 (14)	0.7400 (12)	0.3363 (12)	0.072 (6)*	
H7B1	−0.2535	0.6985	0.2974	0.086*	
H7B2	−0.1883	0.7587	0.4065	0.086*	
C8B	0.2241 (14)	0.7255 (10)	0.4537 (13)	0.072 (6)*	
H8B	0.2438	0.6882	0.3989	0.086*	
C9B	0.3321 (15)	0.7989 (10)	0.4937 (13)	0.071 (6)*	
C10B	0.4343 (15)	0.8091 (11)	0.4515 (13)	0.072 (6)*	
C11B	0.5344 (14)	0.8730 (10)	0.4922 (12)	0.072 (6)*	
H11B	0.6013	0.8796	0.4613	0.087*	
C12B	0.5345 (15)	0.9267 (11)	0.5787 (12)	0.072 (6)*	
H12B	0.6045	0.9683	0.6083	0.086*	
C13B	0.4337 (13)	0.9206 (11)	0.6231 (13)	0.072 (6)*	
H13B	0.4310	0.9599	0.6783	0.086*	
C14B	0.3367 (14)	0.8543 (11)	0.5828 (13)	0.072 (6)*	
H14B	0.2724	0.8464	0.6162	0.087*	
C15B	0.2303 (14)	0.6716 (12)	0.5555 (13)	0.072 (6)*	
C16B	0.3275 (14)	0.5400 (11)	0.6458 (12)	0.072 (6)*	
H16D	0.4071	0.5038	0.6579	0.108*	
H16E	0.2436	0.5076	0.6049	0.108*	
H16F	0.3289	0.5583	0.7190	0.108*	
C17B	−0.1919 (15)	0.8163 (11)	0.2605 (13)	0.072 (6)*	
C18B	−0.0874 (14)	0.8485 (11)	0.2336 (12)	0.072 (6)*	
S2A	0.0232 (4)	0.9234 (3)	0.6166 (4)	0.0572 (13)*	
O3A	0.0642 (12)	0.8444 (8)	0.6881 (9)	0.115 (5)*	
O4A	−0.1156 (11)	0.9512 (8)	0.6065 (10)	0.115 (5)*	
O5A	0.1279 (11)	0.9964 (8)	0.6804 (10)	0.115 (5)*	
H51	0.1260	1.0045	0.7439	0.173*	
O6A	0.0282 (11)	0.9101 (10)	0.5049 (10)	0.115 (4)*	
S2B	−0.0311 (5)	1.0685 (3)	0.8780 (4)	0.0692 (16)*	
O3B	−0.0818 (11)	1.1589 (9)	0.8583 (11)	0.132 (5)*	
O4B	−0.0199 (13)	1.0398 (9)	0.9921 (11)	0.133 (5)*	
O5B	−0.1420 (12)	1.0105 (9)	0.7869 (10)	0.133 (5)*	
H52	−0.1264	1.0087	0.7283	0.200*	
O6B	0.1001 (12)	1.0588 (9)	0.8650 (10)	0.133 (5)*	

### Geometric parameters (Å, °)

**Table:**

Cl1A—C10A	1.721 (17)	O2B—C16B	1.44 (2)
S1A—C2A	1.709 (18)	C2B—C3B	1.359 (19)
S1A—C17A	1.716 (13)	C2B—H2B	0.9313
O1A—C15A	1.18 (2)	C3B—C18B	1.41 (2)
O2A—C15A	1.33 (2)	C3B—H3B	0.9305
O2A—C16A	1.443 (18)	C4B—C18B	1.49 (2)
C2A—C3A	1.357 (17)	C4B—N5B	1.51 (2)
C2A—H2A	0.9313	C4B—H4B1	0.9717
C3A—C18A	1.41 (2)	C4B—H4B2	0.9710
C3A—H3A	0.9304	N5B—C6B	1.511 (19)
C4A—N5A	1.51 (2)	N5B—C8B	1.519 (17)
C4A—C18A	1.522 (16)	N5B—H5B	0.9092
C4A—H4A1	0.9699	C6B—C7B	1.52 (2)
C4A—H4A2	0.9674	C6B—H6B1	0.9690
N5A—C8A	1.508 (15)	C6B—H6B2	0.9711
N5A—C6A	1.51 (2)	C7B—C17B	1.49 (2)
N5A—H5A	0.9102	C7B—H7B1	0.9698
C6A—C7A	1.512 (17)	C7B—H7B2	0.9703
C6A—H6A1	0.9690	C8B—C15B	1.51 (2)
C6A—H6A2	0.9712	C8B—C9B	1.53 (2)
C7A—C17A	1.49 (2)	C8B—H8B	0.9795
C7A—H7A1	0.9694	C9B—C10B	1.38 (3)
C7A—H7A2	0.9697	C9B—C14B	1.40 (2)
C8A—C15A	1.50 (2)	C10B—C11B	1.37 (2)
C8A—C9A	1.52 (2)	C11B—C12B	1.37 (2)
C8A—H8A	0.9803	C11B—H11B	0.9312
C9A—C10A	1.38 (2)	C12B—C13B	1.38 (3)
C9A—C14A	1.40 (2)	C12B—H12B	0.9299
C10A—C11A	1.40 (2)	C13B—C14B	1.38 (2)
C11A—C12A	1.39 (2)	C13B—H13B	0.9301
C11A—H11A	0.9303	C14B—H14B	0.9306
C12A—C13A	1.36 (2)	C16B—H16D	0.9596
C12A—H12A	0.9298	C16B—H16E	0.9613
C13A—C14A	1.38 (2)	C16B—H16F	0.9609
C13A—H13A	0.9307	C17B—C18B	1.36 (2)
C14A—H14A	0.9297	S2A—O6A	1.447 (14)
C16A—H16A	0.9589	S2A—O4A	1.466 (13)
C16A—H16B	0.9591	S2A—O3A	1.470 (13)
C16A—H16C	0.9607	S2A—O5A	1.549 (12)
C17A—C18A	1.35 (2)	O5A—H51	0.8200
Cl1B—C10B	1.733 (18)	S2B—O6B	1.449 (15)
S1B—C2B	1.710 (18)	S2B—O4B	1.468 (15)
S1B—C17B	1.714 (14)	S2B—O3B	1.469 (15)
O1B—C15B	1.18 (2)	S2B—O5B	1.549 (12)
O2B—C15B	1.302 (19)	O5B—H52	0.8200
			
C2A—S1A—C17A	91.5 (7)	C18B—C3B—H3B	124.2
C15A—O2A—C16A	117.1 (12)	C18B—C4B—N5B	110.7 (14)
C3A—C2A—S1A	112.3 (12)	C18B—C4B—H4B1	109.4
C3A—C2A—H2A	123.8	N5B—C4B—H4B1	109.6
S1A—C2A—H2A	123.9	C18B—C4B—H4B2	109.5
C2A—C3A—C18A	111.5 (14)	N5B—C4B—H4B2	109.7
C2A—C3A—H3A	124.3	H4B1—C4B—H4B2	107.9
C18A—C3A—H3A	124.2	C4B—N5B—C6B	109.1 (10)
N5A—C4A—C18A	110.4 (11)	C4B—N5B—C8B	113.2 (13)
N5A—C4A—H4A1	109.4	C6B—N5B—C8B	114.7 (11)
C18A—C4A—H4A1	109.5	C4B—N5B—H5B	106.5
N5A—C4A—H4A2	109.6	C6B—N5B—H5B	106.4
C18A—C4A—H4A2	109.7	C8B—N5B—H5B	106.4
H4A1—C4A—H4A2	108.3	N5B—C6B—C7B	112.3 (13)
C4A—N5A—C8A	112.0 (10)	N5B—C6B—H6B1	109.2
C4A—N5A—C6A	110.4 (11)	C7B—C6B—H6B1	109.2
C8A—N5A—C6A	112.4 (11)	N5B—C6B—H6B2	109.0
C4A—N5A—H5A	107.1	C7B—C6B—H6B2	109.1
C8A—N5A—H5A	107.3	H6B1—C6B—H6B2	107.9
C6A—N5A—H5A	107.2	C17B—C7B—C6B	108.7 (14)
N5A—C6A—C7A	114.3 (13)	C17B—C7B—H7B1	109.9
N5A—C6A—H6A1	108.7	C6B—C7B—H7B1	110.0
C7A—C6A—H6A1	108.7	C17B—C7B—H7B2	109.9
N5A—C6A—H6A2	108.6	C6B—C7B—H7B2	109.9
C7A—C6A—H6A2	108.7	H7B1—C7B—H7B2	108.3
H6A1—C6A—H6A2	107.6	C15B—C8B—N5B	108.6 (13)
C17A—C7A—C6A	108.6 (12)	C15B—C8B—C9B	110.1 (12)
C17A—C7A—H7A1	109.9	N5B—C8B—C9B	110.1 (12)
C6A—C7A—H7A1	110.1	C15B—C8B—H8B	109.3
C17A—C7A—H7A2	109.9	N5B—C8B—H8B	109.3
C6A—C7A—H7A2	110.0	C9B—C8B—H8B	109.4
H7A1—C7A—H7A2	108.4	C10B—C9B—C14B	117.3 (14)
C15A—C8A—N5A	109.6 (11)	C10B—C9B—C8B	122.5 (15)
C15A—C8A—C9A	110.4 (13)	C14B—C9B—C8B	120.0 (16)
N5A—C8A—C9A	108.7 (12)	C11B—C10B—C9B	121.7 (16)
C15A—C8A—H8A	109.3	C11B—C10B—Cl1B	119.3 (14)
N5A—C8A—H8A	109.5	C9B—C10B—Cl1B	119.0 (11)
C9A—C8A—H8A	109.4	C12B—C11B—C10B	119.3 (16)
C10A—C9A—C14A	119.0 (15)	C12B—C11B—H11B	120.4
C10A—C9A—C8A	122.2 (15)	C10B—C11B—H11B	120.3
C14A—C9A—C8A	118.3 (13)	C11B—C12B—C13B	121.7 (14)
C9A—C10A—C11A	123.2 (16)	C11B—C12B—H12B	119.3
C9A—C10A—Cl1A	115.6 (12)	C13B—C12B—H12B	119.0
C11A—C10A—Cl1A	120.8 (11)	C12B—C13B—C14B	117.8 (16)
C12A—C11A—C10A	114.0 (13)	C12B—C13B—H13B	121.1
C12A—C11A—H11A	122.8	C14B—C13B—H13B	121.1
C10A—C11A—H11A	123.2	C13B—C14B—C9B	122.1 (16)
C13A—C12A—C11A	125.4 (16)	C13B—C14B—H14B	118.9
C13A—C12A—H12A	117.3	C9B—C14B—H14B	119.1
C11A—C12A—H12A	117.3	O1B—C15B—O2B	122.7 (16)
C12A—C13A—C14A	118.2 (16)	O1B—C15B—C8B	128.3 (14)
C12A—C13A—H13A	121.0	O2B—C15B—C8B	108.3 (14)
C14A—C13A—H13A	120.8	O2B—C16B—H16D	109.6
C13A—C14A—C9A	119.9 (13)	O2B—C16B—H16E	109.5
C13A—C14A—H14A	120.0	H16D—C16B—H16E	109.4
C9A—C14A—H14A	120.1	O2B—C16B—H16F	109.6
O1A—C15A—O2A	124.9 (15)	H16D—C16B—H16F	109.4
O1A—C15A—C8A	125.7 (15)	H16E—C16B—H16F	109.3
O2A—C15A—C8A	109.3 (14)	C18B—C17B—C7B	125.1 (13)
O2A—C16A—H16A	109.5	C18B—C17B—S1B	110.7 (12)
O2A—C16A—H16B	109.4	C7B—C17B—S1B	123.0 (12)
H16A—C16A—H16B	109.6	C17B—C18B—C3B	113.9 (13)
O2A—C16A—H16C	109.3	C17B—C18B—C4B	122.3 (14)
H16A—C16A—H16C	109.5	C3B—C18B—C4B	123.7 (15)
H16B—C16A—H16C	109.5	O6A—S2A—O4A	111.8 (7)
C18A—C17A—C7A	123.8 (12)	O6A—S2A—O3A	111.7 (8)
C18A—C17A—S1A	111.0 (12)	O4A—S2A—O3A	109.3 (8)
C7A—C17A—S1A	124.1 (11)	O6A—S2A—O5A	108.6 (8)
C17A—C18A—C3A	113.7 (11)	O4A—S2A—O5A	107.7 (7)
C17A—C18A—C4A	123.6 (14)	O3A—S2A—O5A	107.6 (6)
C3A—C18A—C4A	122.7 (13)	S2A—O5A—H51	109.5
C2B—S1B—C17B	91.7 (8)	O6B—S2B—O4B	111.6 (7)
C15B—O2B—C16B	117.0 (13)	O6B—S2B—O3B	111.8 (8)
C3B—C2B—S1B	112.1 (12)	O4B—S2B—O3B	109.5 (8)
C3B—C2B—H2B	123.8	O6B—S2B—O5B	108.6 (8)
S1B—C2B—H2B	124.0	O4B—S2B—O5B	107.6 (8)
C2B—C3B—C18B	111.5 (15)	O3B—S2B—O5B	107.6 (7)
C2B—C3B—H3B	124.3	S2B—O5B—H52	109.5

### Hydrogen-bond geometry (Å, °)

**Table:**

*D*—H···*A*	*D*—H	H···*A*	*D*···*A*	*D*—H···*A*
N5A—H5A···O3A	0.91	1.91	2.785 (16)	161
N5B—H5B···O6A	0.91	1.94	2.795 (19)	157
O5A—H51···O6B	0.82	1.85	2.640 (17)	161
O5B—H52···O4A	0.82	1.82	2.567 (17)	152
C4A—H4A1···O4B^i^	0.97	2.35	3.17 (2)	142
C4A—H4A2···O1B	0.97	2.52	3.225 (17)	129
C3B—H3B···O4B^ii^	0.93	2.41	3.28 (2)	154
C6A—H6A2···O3B^i^	0.97	2.31	3.175 (19)	149
C4B—H4B2···O3B^iii^	0.97	2.23	3.13 (2)	154

Symmetry codes: (i) −*x*, *y*−1/2, −*z*+2; (ii) *x*, *y*, *z*−1; (iii) −*x*, *y*−1/2, −*z*+1.
